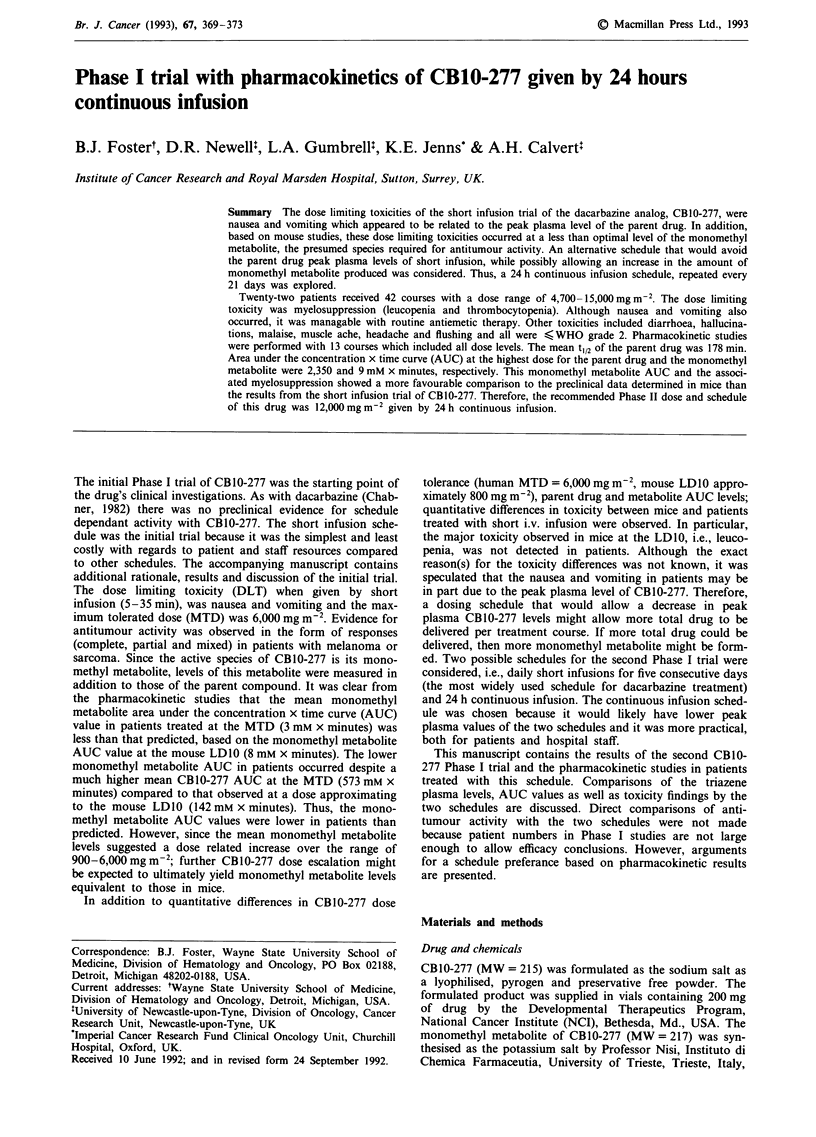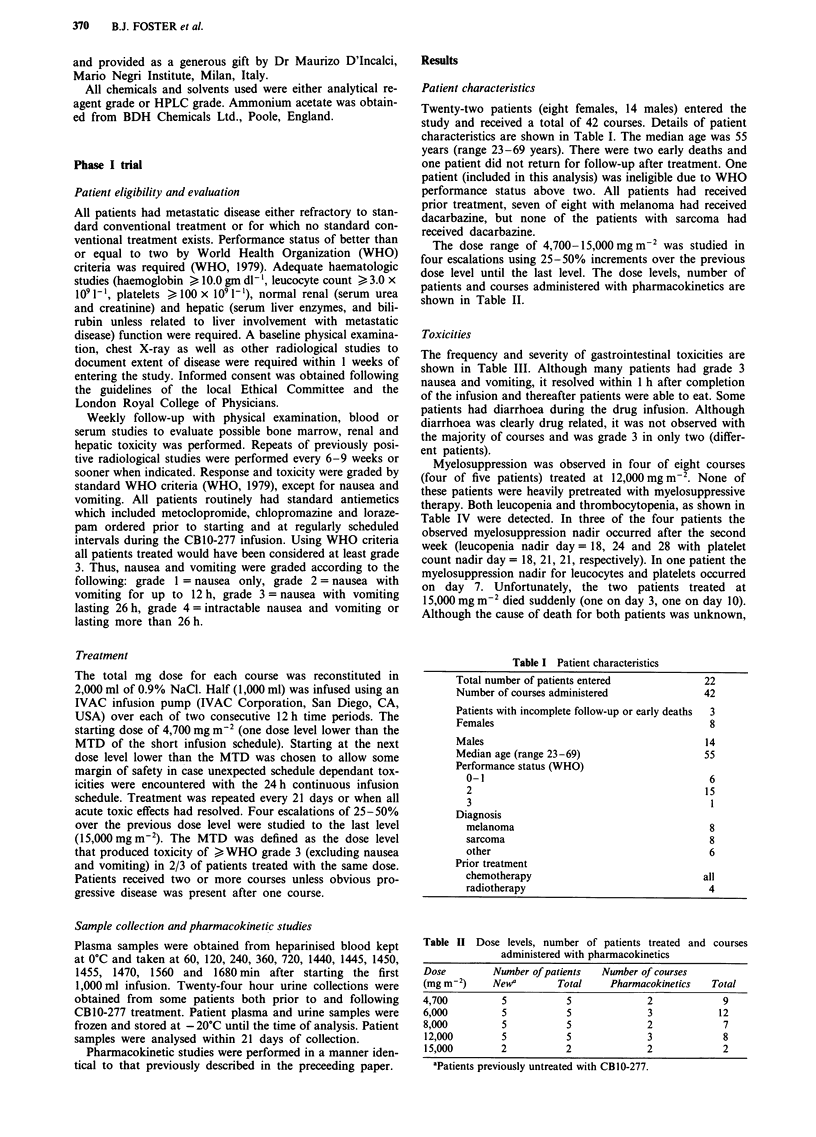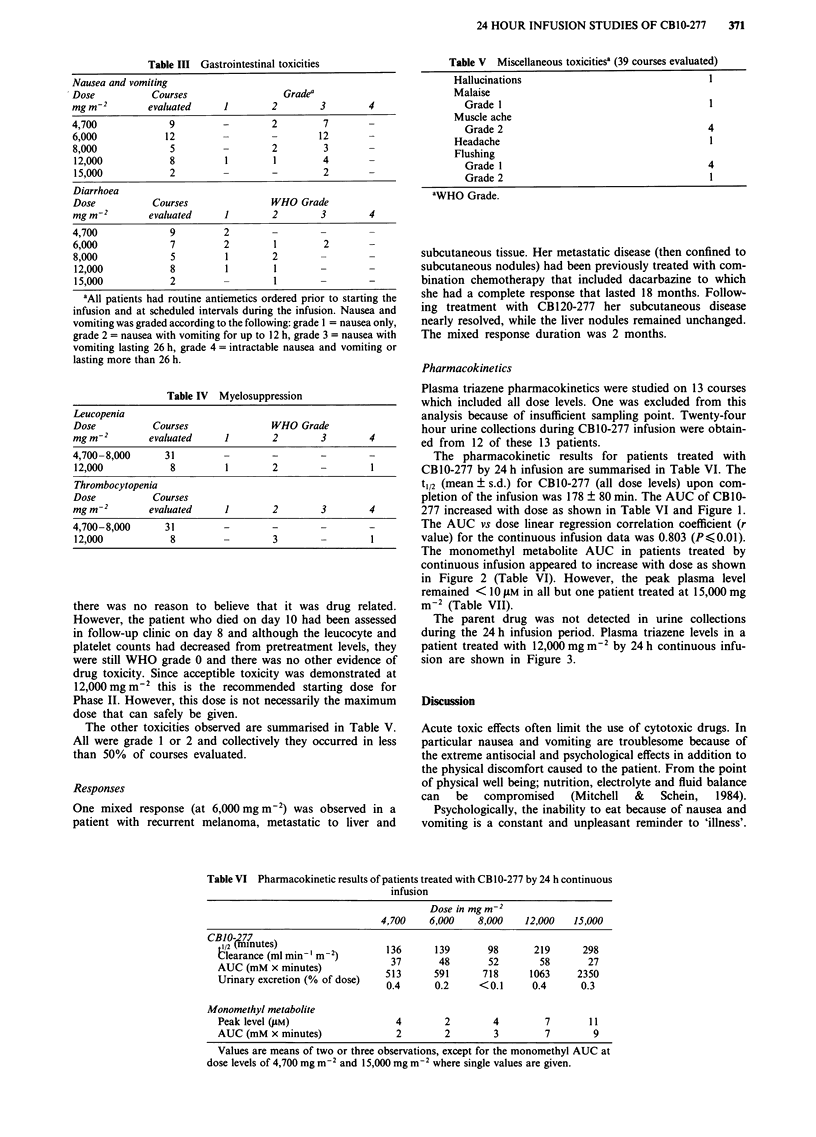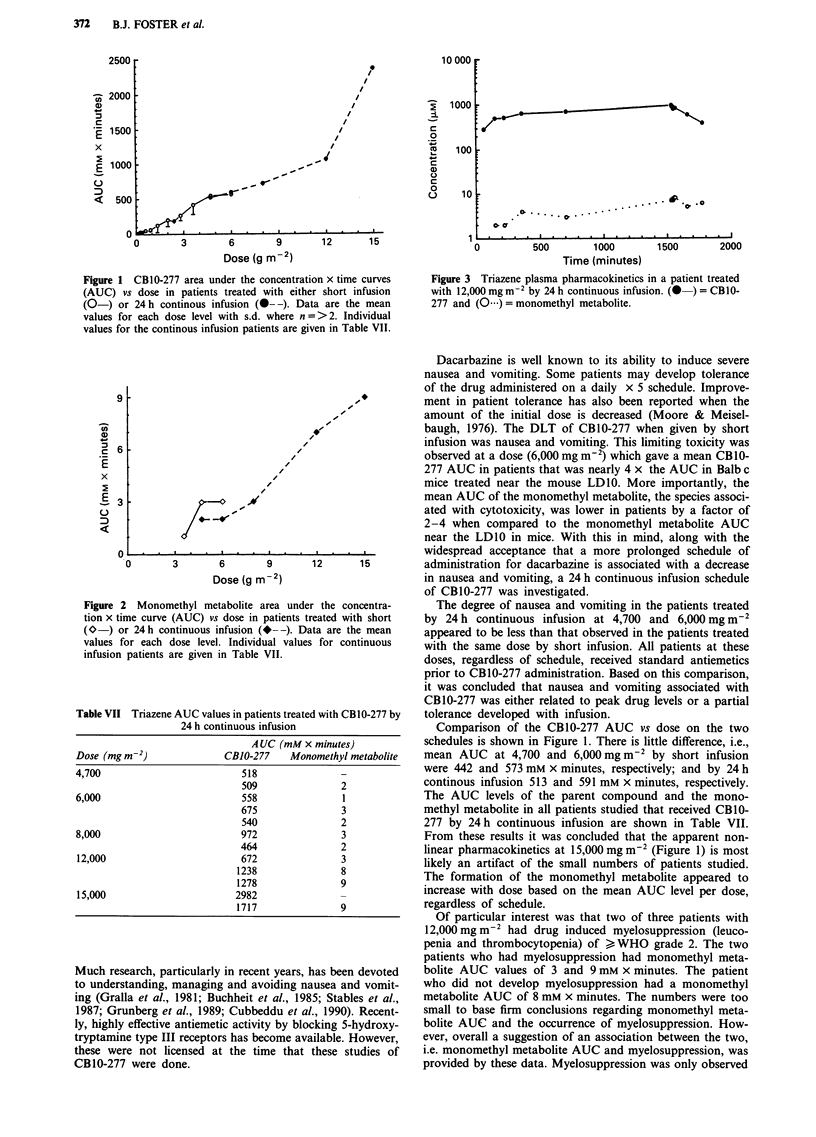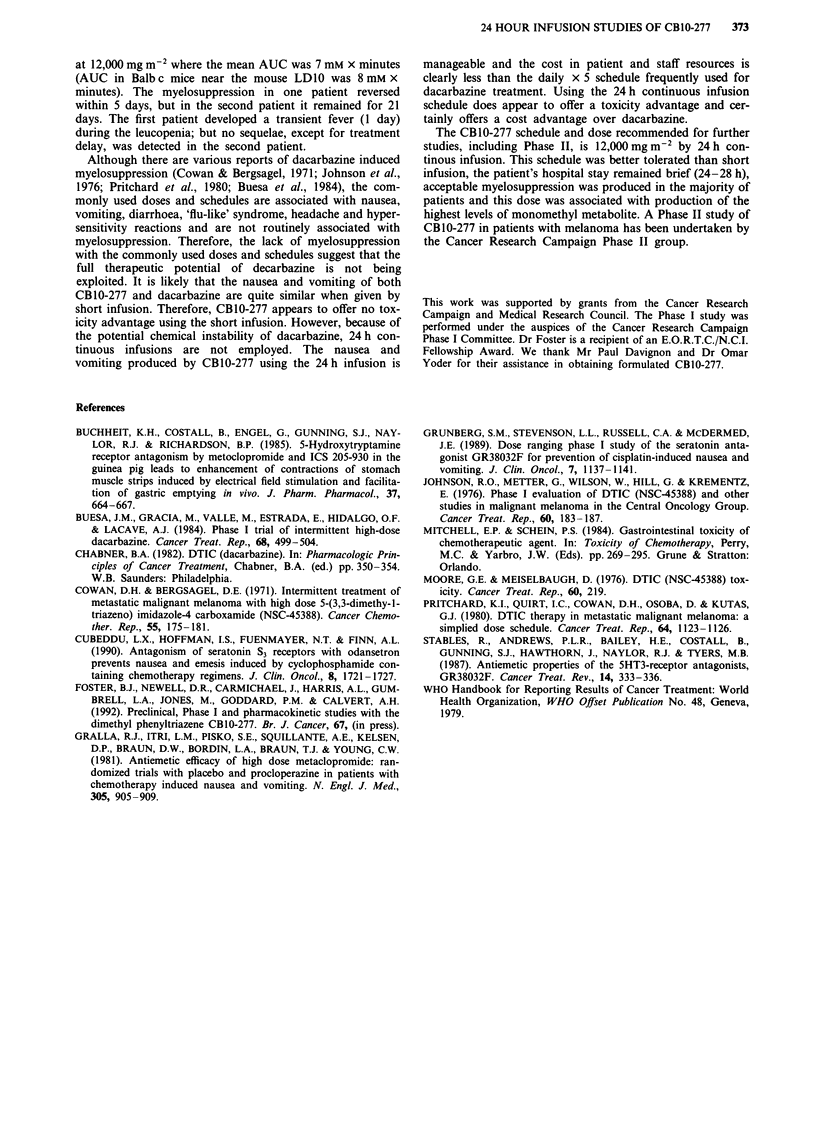# Phase I trial with pharmacokinetics of CB10-277 given by 24 hours continuous infusion.

**DOI:** 10.1038/bjc.1993.67

**Published:** 1993-02

**Authors:** B. J. Foster, D. R. Newell, L. A. Gumbrell, K. E. Jenns, A. H. Calvert

**Affiliations:** Institute of Cancer Research, Sutton, Surrey, UK.

## Abstract

The dose limiting toxicities of the short infusion trial of the dacarbazine analog, CB10-277, were nausea and vomiting which appeared to be related to the peak plasma level of the parent drug. In addition, based on mouse studies, these dose limiting toxicities occurred at a less than optimal level of the monomethyl metabolite, the presumed species required for antitumour activity. An alternative schedule that would avoid the parent drug peak plasma levels of short infusion, while possibly allowing an increase in the amount of monomethyl metabolite produced was considered. Thus, a 24 h continuous infusion schedule, repeated every 21 days was explored. Twenty-two patients received 42 courses with a dose range of 4,700-15,000 mg m-2. The dose limiting toxicity was myelosuppression (leucopenia and thrombocytopenia). Although nausea and vomiting also occurred, it was manageable with routine antiemetic therapy. Other toxicities included diarrhoea, hallucinations, malaise, muscle ache, headache and flushing and all were < or = WHO grade 2. Pharmacokinetic studies were performed with 13 courses which included all dose levels. The mean t1/2 of the parent drug was 178 min. Area under the concentration x time curve (AUC) at the highest dose for the parent drug and the monomethyl metabolite were 2,350 and 9 mM x minutes, respectively. This monomethyl metabolite AUC and the associated myelosuppression showed a more favourable comparison to the preclinical data determined in mice than the results from the short infusion trial of CB10-277. Therefore, the recommended Phase II dose and schedule of this drug was 12,000 mg m-2 given by 24 h continuous infusion.


Br. J. Cancer (1993), 67, 369-373 © Macmillan Press Ltd., 1993

### Phase I trial with pharmacokinetics of CB10-277 given by 24 hours continuous infusion

B.J. Foster[†], D.R. Newell[‡], L.A. Gumbrell[‡], K.E. Jenns[*] & A.H. Calvert[‡]

*Institute of Cancer Research and Royal Marsden Hospital, Sutton, Surrey, UK.*

**Summary** The dose limiting toxicities of the short infusion trial of the dacarbazine analog, CB10-277, were nausea and vomiting which appeared to be related to the peak plasma level of the parent drug. In addition, based on mouse studies, these dose limiting toxicities occurred at a less than optimal level of the monomethyl metabolite, the presumed species required for antitumour activity. An alternative schedule that would avoid the parent drug peak plasma levels of short infusion, while possibly allowing an increase in the amount of monomethyl metabolite produced was considered. Thus, a 24 h continuous infusion schedule, repeated every 21 days was explored.

Twenty-two patients received 42 courses with a dose range of 4,700-15,000 mg m$^{-2}$. The dose limiting toxicity was myelosuppression (leucopenia and thrombocytopenia). Although nausea and vomiting also occurred, it was managable with routine antiemetic therapy. Other toxicities included diarrhoea, hallucinations, malaise, muscle ache, headache and flushing and all were $\leqslant$WHO grade 2. Pharmacokinetic studies were performed with 13 courses which included all dose levels. The mean $t_{1/2}$ of the parent drug was 178 min. Area under the concentration × time curve (AUC) at the highest dose for the parent drug and the monomethyl metabolite were 2,350 and 9 mM × minutes, respectively. This monomethyl metabolite AUC and the associated myelosuppression showed a more favourable comparison to the preclinical data determined in mice than the results from the short infusion trial of CB10-277. Therefore, the recommended Phase II dose and schedule of this drug was 12,000 mg m$^{-2}$ given by 24 h continuous infusion.

The initial Phase I trial of CB10-277 was the starting point of the drug's clinical investigations. As with dacarbazine (Chabner, 1982) there was no preclinical evidence for schedule dependant activity with CB10-277. The short infusion schedule was the initial trial because it was the simplest and least costly with regards to patient and staff resources compared to other schedules. The accompanying manuscript contains additional rationale, results and discussion of the initial trial. The dose limiting toxicity (DLT) when given by short infusion (5-35 min), was nausea and vomiting and the maximum tolerated dose (MTD) was 6,000 mg m$^{-2}$. Evidence for antitumour activity was observed in the form of responses (complete, partial and mixed) in patients with melanoma or sarcoma. Since the active species of CB10-277 is its monomethyl metabolite, levels of this metabolite were measured in addition to those of the parent compound. It was clear from the pharmacokinetic studies that the mean monomethyl metabolite area under the concentration × time curve (AUC) value in patients treated at the MTD (3 mM × minutes) was less than that predicted, based on the monomethyl metabolite AUC value at the mouse LD10 (8 mM × minutes). The lower monomethyl metabolite AUC in patients occurred despite a much higher mean CB10-277 AUC at the MTD (573 mM × minutes) compared to that observed at a dose approximating to the mouse LD10 (142 mM × minutes). Thus, the monomethyl metabolite AUC values were lower in patients than predicted. However, since the mean monomethyl metabolite levels suggested a dose related increase over the range of 900-6,000 mg m$^{-2}$; further CB10-277 dose escalation might be expected to ultimately yield monomethyl metabolite levels equivalent to those in mice.

In addition to quantitative differences in CB10-277 dose tolerance (human MTD = 6,000 mg m$^{-2}$, mouse LD10 approximately 800 mg m$^{-2}$), parent drug and metabolite AUC levels; quantitative differences in toxicity between mice and patients treated with short i.v. infusion were observed. In particular, the major toxicity observed in mice at the LD10, i.e., leucopenia, was not detected in patients. Although the exact reason(s) for the toxicity differences was not known, it was speculated that the nausea and vomiting in patients may be in part due to the peak plasma level of CB10-277. Therefore, a dosing schedule that would allow a decrease in peak plasma CB10-277 levels might allow more total drug to be delivered per treatment course. If more total drug could be delivered, then more monomethyl metabolite might be formed. Two possible schedules for the second Phase I trial were considered, i.e., daily short infusions for five consecutive days (the most widely used schedule for dacarbazine treatment) and 24 h continuous infusion. The continuous infusion schedule was chosen because it would likely have lower peak plasma values of the two schedules and it was more practical, both for patients and hospital staff.

This manuscript contains the results of the second CB10-277 Phase I trial and the pharmacokinetic studies in patients treated with this schedule. Comparisons of the triazene plasma levels, AUC values as well as toxicity findings by the two schedules are discussed. Direct comparisons of antitumour activity with the two schedules were not made because patient numbers in Phase I studies are not large enough to allow efficacy conclusions. However, arguments for a schedule preferance based on pharmacokinetic results are presented.

Correspondence: B.J. Foster, Wayne State University School of Medicine, Division of Hematology and Oncology, PO Box 02188, Detroit, Michigan 48202-0188, USA.

Current addresses: [†]Wayne State University School of Medicine, Division of Hematology and Oncology, Detroit, Michigan, USA. [‡]University of Newcastle-upon-Tyne, Division of Oncology, Cancer Research Unit, Newcastle-upon-Tyne, UK

[*]Imperial Cancer Research Fund Clinical Oncology Unit, Churchill Hospital, Oxford, UK.

Received 10 June 1992; and in revised form 24 September 1992.

### Materials and methods

### *Drug and chemicals*

CB10-277 (MW = 215) was formulated as the sodium salt as a lyophilised, pyrogen and preservative free powder. The formulated product was supplied in vials containing 200 mg of drug by the Developmental Therapeutics Program, National Cancer Institute (NCI), Bethesda, Md., USA. The monomethyl metabolite of CB10-277 (MW = 217) was synthesised as the potassium salt by Professor Nisi, Instituto di Chemica Farmaceutia, University of Trieste, Trieste, Italy, and provided as a generous gift by Dr Maurizo D'Incalci, Mario Negri Institute, Milan, Italy.

All chemicals and solvents used were either analytical reagent grade or HPLC grade. Ammonium acetate was obtained from BDH Chemicals Ltd., Poole, England.

### Phase I trial

*Patient eligibility and evaluation*

All patients had metastatic disease either refractory to standard conventional treatment or for which no standard conventional treatment exists. Performance status of better than or equal to two by World Health Organization (WHO) criteria was required (WHO, 1979). Adequate haematologic studies (haemoglobin $\geqslant 10.0$ gm $dl^{-1}$, leucocyte count $\geqslant 3.0 \times 10^9 l^{-1}$, platelets $\geqslant 100 \times 10^9 l^{-1}$), normal renal (serum urea and creatinine) and hepatic (serum liver enzymes, and bilirubin unless related to liver involvement with metastatic disease) function were required. A baseline physical examination, chest X-ray as well as other radiological studies to document extent of disease were required within 1 weeks of entering the study. Informed consent was obtained following the guidelines of the local Ethical Committee and the London Royal College of Physicians.

Weekly follow-up with physical examination, blood or serum studies to evaluate possible bone marrow, renal and hepatic toxicity was performed. Repeats of previously positive radiological studies were performed every 6–9 weeks or sooner when indicated. Response and toxicity were graded by standard WHO criteria (WHO, 1979), except for nausea and vomiting. All patients routinely had standard antiemetics which included metoclopromide, chlopromazine and lorazepam ordered prior to starting and at regularly scheduled intervals during the CB10-277 infusion. Using WHO criteria all patients treated would have been considered at least grade 3. Thus, nausea and vomiting were graded according to the following: grade 1 = nausea only, grade 2 = nausea with vomiting for up to 12 h, grade 3 = nausea with vomiting lasting 26 h, grade 4 = intractable nausea and vomiting or lasting more than 26 h.

*Treatment*

The total mg dose for each course was reconstituted in 2,000 ml of 0.9% NaCl. Half (1,000 ml) was infused using an IVAC infusion pump (IVAC Corporation, San Diego, CA, USA) over each of two consecutive 12 h time periods. The starting dose of 4,700 mg $m^{-2}$ (one dose level lower than the MTD of the short infusion schedule). Starting at the next dose level lower than the MTD was chosen to allow some margin of safety in case unexpected schedule dependant toxicities were encountered with the 24 h continuous infusion schedule. Treatment was repeated every 21 days or when all acute toxic effects had resolved. Four escalations of 25–50% over the previous dose level were studied to the last level (15,000 mg $m^{-2}$). The MTD was defined as the dose level that produced toxicity of $\geqslant$WHO grade 3 (excluding nausea and vomiting) in 2/3 of patients treated with the same dose. Patients received two or more courses unless obvious progressive disease was present after one course.

*Sample collection and pharmacokinetic studies*

Plasma samples were obtained from heparinised blood kept at 0°C and taken at 60, 120, 240, 360, 720, 1440, 1445, 1450, 1455, 1470, 1560 and 1680 min after starting the first 1,000 ml infusion. Twenty-four hour urine collections were obtained from some patients both prior to and following CB10-277 treatment. Patient plasma and urine samples were frozen and stored at −20°C until the time of analysis. Patient samples were analysed within 21 days of collection.

Pharmacokinetic studies were performed in a manner identical to that previously described in the preceeding paper.

### Results

*Patient characteristics*

Twenty-two patients (eight females, 14 males) entered the study and received a total of 42 courses. Details of patient characteristics are shown in Table I. The median age was 55 years (range 23–69 years). There were two early deaths and one patient did not return for follow-up after treatment. One patient (included in this analysis) was ineligible due to WHO performance status above two. All patients had received prior treatment, seven of eight with melanoma had received dacarbazine, but none of the patients with sarcoma had received dacarbazine.

The dose range of 4,700–15,000 mg $m^{-2}$ was studied in four escalations using 25–50% increments over the previous dose level until the last level. The dose levels, number of patients and courses administered with pharmacokinetics are shown in Table II.

*Toxicities*

The frequency and severity of gastrointestinal toxicities are shown in Table III. Although many patients had grade 3 nausea and vomiting, it resolved within 1 h after completion of the infusion and thereafter patients were able to eat. Some patients had diarrhoea during the drug infusion. Although diarrhoea was clearly drug related, it was not observed with the majority of courses and was grade 3 in only two (different patients).

Myelosuppression was observed in four of eight courses (four of five patients) treated at 12,000 mg $m^{-2}$. None of these patients were heavily pretreated with myelosuppressive therapy. Both leucopenia and thrombocytopenia, as shown in Table IV were detected. In three of the four patients the observed myelosuppression nadir occurred after the second week (leucopenia nadir day = 18, 24 and 28 with platelet count nadir day = 18, 21, 21, respectively). In one patient the myelosuppression nadir for leucocytes and platelets occurred on day 7. Unfortunately, the two patients treated at 15,000 mg $m^{-2}$ died suddenly (one on day 3, one on day 10). Although the cause of death for both patients was unknown,

**Table I** Patient characteristics

| | |
|---|---|
| Total number of patients entered | 22 |
| Number of courses administered | 42 |
| Patients with incomplete follow-up or early deaths | 3 |
| Females | 8 |
| Males | 14 |
| Median age (range 23–69) | 55 |
| Performance status (WHO) | |
| 0–1 | 6 |
| 2 | 15 |
| 3 | 1 |
| Diagnosis | |
| melanoma | 8 |
| sarcoma | 8 |
| other | 6 |
| Prior treatment | |
| chemotherapy | all |
| radiotherapy | 4 |

**Table II** Dose levels, number of patients treated and courses administered with pharmacokinetics

| *Dose* ($mg\,m^{-2}$) | *Number of patients* *New*[a] | *Total* | *Number of courses* *Pharmacokinetics* | *Total* |
|---|---|---|---|---|
| 4,700 | 5 | 5 | 2 | 9 |
| 6,000 | 5 | 5 | 3 | 12 |
| 8,000 | 5 | 5 | 2 | 7 |
| 12,000 | 5 | 5 | 3 | 8 |
| 15,000 | 2 | 2 | 2 | 2 |

[a]Patients previously untreated with CB10-277.

**Table III** Gastrointestinal toxicities

| *Nausea and vomiting* | | | | | |
|---|---|---|---|---|---|
| *Dose* | *Courses* | | *Grade*[a] | | |
| *mg m*$^{-2}$ | *evaluated* | *1* | *2* | *3* | *4* |
| 4,700 | 9 | – | 2 | 7 | – |
| 6,000 | 12 | – | – | 12 | – |
| 8,000 | 5 | – | 2 | 3 | – |
| 12,000 | 8 | 1 | 1 | 4 | – |
| 15,000 | 2 | – | – | 2 | – |
| *Diarrhoea* | | | | | |
| *Dose* | *Courses* | | *WHO Grade* | | |
| *mg m*$^{-2}$ | *evaluated* | *1* | *2* | *3* | *4* |
| 4,700 | 9 | 2 | – | – | – |
| 6,000 | 7 | 2 | 1 | 2 | – |
| 8,000 | 5 | 1 | 2 | – | – |
| 12,000 | 8 | 1 | 1 | – | – |
| 15,000 | 2 | – | 1 | – | – |

[a]All patients had routine antiemetics ordered prior to starting the infusion and at scheduled intervals during the infusion. Nausea and vomiting was graded according to the following: grade 1 = nausea only, grade 2 = nausea with vomiting for up to 12 h, grade 3 = nausea with vomiting lasting 26 h, grade 4 = intractable nausea and vomiting or lasting more than 26 h.

**Table IV** Myelosuppression

| *Leucopenia* | | | | | |
|---|---|---|---|---|---|
| *Dose* | *Courses* | | *WHO Grade* | | |
| *mg m*$^{-2}$ | *evaluated* | *1* | *2* | *3* | *4* |
| 4,700–8,000 | 31 | – | – | – | – |
| 12,000 | 8 | 1 | 2 | – | 1 |
| *Thrombocytopenia* | | | | | |
| *Dose* | *Courses* | | | | |
| *mg m*$^{-2}$ | *evaluated* | *1* | *2* | *3* | *4* |
| 4,700–8,000 | 31 | – | – | – | – |
| 12,000 | 8 | – | 3 | – | 1 |

there was no reason to believe that it was drug related. However, the patient who died on day 10 had been assessed in follow-up clinic on day 8 and although the leucocyte and platelet counts had decreased from pretreatment levels, they were still WHO grade 0 and there was no other evidence of drug toxicity. Since acceptible toxicity was demonstrated at 12,000 mg m$^{-2}$ this is the recommended starting dose for Phase II. However, this dose is not necessarily the maximum dose that can safely be given.

The other toxicities observed are summarised in Table V. All were grade 1 or 2 and collectively they occurred in less than 50% of courses evaluated.

*Responses*

One mixed response (at 6,000 mg m$^{-2}$) was observed in a patient with recurrent melanoma, metastatic to liver and subcutaneous tissue. Her metastatic disease (then confined to subcutaneous nodules) had been previously treated with combination chemotherapy that included dacarbazine to which she had a complete response that lasted 18 months. Following treatment with CB120-277 her subcutaneous disease nearly resolved, while the liver nodules remained unchanged. The mixed response duration was 2 months.

**Table V** Miscellaneous toxicities[a] (39 courses evaluated)

| | |
|---|---|
| Hallucinations | 1 |
| Malaise | |
| Grade 1 | 1 |
| Muscle ache | |
| Grade 2 | 4 |
| Headache | 1 |
| Flushing | |
| Grade 1 | 4 |
| Grade 2 | 1 |

[a]WHO Grade.

*Pharmacokinetics*

Plasma triazene pharmacokinetics were studied on 13 courses which included all dose levels. One was excluded from this analysis because of insufficient sampling point. Twenty-four hour urine collections during CB10-277 infusion were obtained from 12 of these 13 patients.

The pharmacokinetic results for patients treated with CB10-277 by 24 h infusion are summarised in Table VI. The $t_{1/2}$ (mean ± s.d.) for CB10-277 (all dose levels) upon completion of the infusion was 178 ± 80 min. The AUC of CB10-277 increased with dose as shown in Table VI and Figure 1. The AUC *vs* dose linear regression correlation coefficient (*r* value) for the continuous infusion data was 0.803 ($P \leqslant 0.01$). The monomethyl metabolite AUC in patients treated by continuous infusion appeared to increase with dose as shown in Figure 2 (Table VI). However, the peak plasma level remained $< 10\ \mu M$ in all but one patient treated at 15,000 mg m$^{-2}$ (Table VII).

The parent drug was not detected in urine collections during the 24 h infusion period. Plasma triazene levels in a patient treated with 12,000 mg m$^{-2}$ by 24 h continuous infusion are shown in Figure 3.

### Discussion

Acute toxic effects often limit the use of cytotoxic drugs. In particular nausea and vomiting are troublesome because of the extreme antisocial and psychological effects in addition to the physical discomfort caused to the patient. From the point of physical well being; nutrition, electrolyte and fluid balance can be compromised (Mitchell & Schein, 1984). Psychologically, the inability to eat because of nausea and vomiting is a constant and unpleasant reminder to 'illness'.

**Table VI** Pharmacokinetic results of patients treated with CB10-277 by 24 h continuous infusion

| | | | *Dose in mg m*$^{-2}$ | | |
|---|---|---|---|---|---|
| | *4,700* | *6,000* | *8,000* | *12,000* | *15,000* |
| *CB10-277* | | | | | |
| $t_{1/2}$ (minutes) | 136 | 139 | 98 | 219 | 298 |
| Clearance (ml min$^{-1}$ m$^{-2}$) | 37 | 48 | 52 | 58 | 27 |
| AUC (mM × minutes) | 513 | 591 | 718 | 1063 | 2350 |
| Urinary excretion (% of dose) | 0.4 | 0.2 | <0.1 | 0.4 | 0.3 |
| *Monomethyl metabolite* | | | | | |
| Peak level (μM) | 4 | 2 | 4 | 7 | 11 |
| AUC (mM × minutes) | 2 | 2 | 3 | 7 | 9 |

Values are means of two or three observations, except for the monomethyl AUC at dose levels of 4,700 mg m$^{-2}$ and 15,000 mg m$^{-2}$ where single values are given.

**Figure 1** CB10-277 area under the concentration × time curves (AUC) *vs* dose in patients treated with either short infusion (O—) or 24 h continous infusion (●– –). Data are the mean values for each dose level with s.d. where $n = > 2$. Individual values for the continous infusion patients are given in Table VII.

**Figure 2** Monomethyl metabolite area under the concentration × time curve (AUC) *vs* dose in patients treated with short (◇—) or 24 h continuous infusion (◆– –). Data are the mean values for each dose level. Individual values for continuous infusion patients are given in Table VII.

**Table VII** Triazene AUC values in patients treated with CB10-277 by 24 h continuous infusion

| | *AUC (mM × minutes)* | |
|---|---|---|
| *Dose (mg m⁻²)* | *CB10-277* | *Monomethyl metabolite* |
| 4,700 | 518 | – |
| | 509 | 2 |
| 6,000 | 558 | 1 |
| | 675 | 3 |
| | 540 | 2 |
| 8,000 | 972 | 3 |
| | 464 | 2 |
| 12,000 | 672 | 3 |
| | 1238 | 8 |
| | 1278 | 9 |
| 15,000 | 2982 | – |
| | 1717 | 9 |

Much research, particularly in recent years, has been devoted to understanding, managing and avoiding nausea and vomiting (Gralla *et al.*, 1981; Buchheit *et al.*, 1985; Stables *et al.*, 1987; Grunberg *et al.*, 1989; Cubbeddu *et al.*, 1990). Recently, highly effective antiemetic activity by blocking 5-hydroxytryptamine type III receptors has become available. However, these were not licensed at the time that these studies of CB10-277 were done.

**Figure 3** Triazene plasma pharmacokinetics in a patient treated with 12,000 mg m⁻² by 24 h continuous infusion. (●—) = CB10-277 and (O···) = monomethyl metabolite.

Dacarbazine is well known to its ability to induce severe nausea and vomiting. Some patients may develop tolerance of the drug administered on a daily × 5 schedule. Improvement in patient tolerance has also been reported when the amount of the initial dose is decreased (Moore & Meiselbaugh, 1976). The DLT of CB10-277 when given by short infusion was nausea and vomiting. This limiting toxicity was observed at a dose (6,000 mg m⁻²) which gave a mean CB10-277 AUC in patients that was nearly 4 × the AUC in Balb c mice treated near the mouse LD10. More importantly, the mean AUC of the monomethyl metabolite, the species associated with cytotoxicity, was lower in patients by a factor of 2–4 when compared to the monomethyl metabolite AUC near the LD10 in mice. With this in mind, along with the widespread acceptance that a more prolonged schedule of administration for dacarbazine is associated with a decrease in nausea and vomiting, a 24 h continuous infusion schedule of CB10-277 was investigated.

The degree of nausea and vomiting in the patients treated by 24 h continuous infusion at 4,700 and 6,000 mg m⁻² appeared to be less than that observed in the patients treated with the same dose by short infusion. All patients at these doses, regardless of schedule, received standard antiemetics prior to CB10-277 administration. Based on this comparison, it was concluded that nausea and vomiting associated with CB10-277 was either related to peak drug levels or a partial tolerance developed with infusion.

Comparison of the CB10-277 AUC *vs* dose on the two schedules is shown in Figure 1. There is little difference, i.e., mean AUC at 4,700 and 6,000 mg m⁻² by short infusion were 442 and 573 mM × minutes, respectively; and by 24 h continous infusion 513 and 591 mM × minutes, respectively. The AUC levels of the parent compound and the monomethyl metabolite in all patients studied that received CB10-277 by 24 h continuous infusion are shown in Table VII. From these results it was concluded that the apparent nonlinear pharmacokinetics at 15,000 mg m⁻² (Figure 1) is most likely an artifact of the small numbers of patients studied. The formation of the monomethyl metabolite appeared to increase with dose based on the mean AUC level per dose, regardless of schedule.

Of particular interest was that two of three patients with 12,000 mg m⁻² had drug induced myelosuppression (leucopenia and thrombocytopenia) of ≥ WHO grade 2. The two patients who had myelosuppression had monomethyl metabolite AUC values of 3 and 9 mM × minutes. The patient who did not develop myelosuppression had a monomethyl metabolite AUC of 8 mM × minutes. The numbers were too small to base firm conclusions regarding monomethyl metabolite AUC and the occurrence of myelosuppression. However, overall a suggestion of an association between the two, i.e. monomethyl metabolite AUC and myelosuppression, was provided by these data. Myelosuppression was only observed at 12,000 mg $m^{-2}$ where the mean AUC was 7 mM × minutes (AUC in Balb c mice near the mouse LD10 was 8 mM × minutes). The myelosuppression in one patient reversed within 5 days, but in the second patient it remained for 21 days. The first patient developed a transient fever (1 day) during the leucopenia; but no sequelae, except for treatment delay, was detected in the second patient.

Although there are various reports of dacarbazine induced myelosuppression (Cowan & Bergsagel, 1971; Johnson *et al.*, 1976; Pritchard *et al.*, 1980; Buesa *et al.*, 1984), the commonly used doses and schedules are associated with nausea, vomiting, diarrhoea, 'flu-like' syndrome, headache and hypersensitivity reactions and are not routinely associated with myelosuppression. Therefore, the lack of myelosuppression with the commonly used doses and schedules suggest that the full therapeutic potential of decarbazine is not being exploited. It is likely that the nausea and vomiting of both CB10-277 and dacarbazine are quite similar when given by short infusion. Therefore, CB10-277 appears to offer no toxicity advantage using the short infusion. However, because of the potential chemical instability of dacarbazine, 24 h continuous infusions are not employed. The nausea and vomiting produced by CB10-277 using the 24 h infusion is manageable and the cost in patient and staff resources is clearly less than the daily × 5 schedule frequently used for dacarbazine treatment. Using the 24 h continuous infusion schedule does appear to offer a toxicity advantage and certainly offers a cost advantage over dacarbazine.

The CB10-277 schedule and dose recommended for further studies, including Phase II, is 12,000 mg $m^{-2}$ by 24 h continous infusion. This schedule was better tolerated than short infusion, the patient's hospital stay remained brief (24–28 h), acceptable myelosuppression was produced in the majority of patients and this dose was associated with production of the highest levels of monomethyl metabolite. A Phase II study of CB10-277 in patients with melanoma has been undertaken by the Cancer Research Campaign Phase II group.

This work was supported by grants from the Cancer Research Campaign and Medical Research Council. The Phase I study was performed under the auspices of the Cancer Research Campaign Phase I Committee. Dr Foster is a recipient of an E.O.R.T.C./N.C.I. Fellowship Award. We thank Mr Paul Davignon and Dr Omar Yoder for their assistance in obtaining formulated CB10-277.

### References

BUCHHEIT, K.H., COSTALL, B., ENGEL, G., GUNNING, S.J., NAYLOR, R.J. & RICHARDSON, B.P. (1985). 5-Hydroxytryptamine receptor antagonism by metoclopromide and ICS 205-930 in the guinea pig leads to enhancement of contractions of stomach muscle strips induced by electrical field stimulation and facilitation of gastric emptying *in vivo*. *J. Pharm. Pharmacol.*, **37**, 664–667.

BUESA, J.M., GRACIA, M., VALLE, M., ESTRADA, E., HIDALGO, O.F. & LACAVE, A.J. (1984). Phase I trial of intermittent high-dose dacarbazine. *Cancer Treat. Rep.*, **68**, 499–504.

CHABNER, B.A. (1982). DTIC (dacarbazine). In: *Pharmacologic Principles of Cancer Treatment*, Chabner, B.A. (ed.) pp. 350–354. W.B. Saunders: Philadelphia.

COWAN, D.H. & BERGSAGEL, D.E. (1971). Intermittent treatment of metastatic malignant melanoma with high dose 5-(3,3-dimethy-1-triazeno) imidazole-4 carboxamide (NSC-45388). *Cancer Chemother. Rep.*, **55**, 175–181.

CUBEDDU, L.X., HOFFMAN, I.S., FUENMAYER, N.T. & FINN, A.L. (1990). Antagonism of seratonin $S_3$ receptors with odansetron prevents nausea and emesis induced by cyclophosphamide containing chemotherapy regimens. *J. Clin. Oncol.*, **8**, 1721–1727.

FOSTER, B.J., NEWELL, D.R., CARMICHAEL, J., HARRIS, A.L., GUMBRELL, L.A., JONES, M., GODDARD, P.M. & CALVERT, A.H. (1992). Preclinical, Phase I and pharmacokinetic studies with the dimethyl phenyltriazene CB10-277. *Br. J. Cancer*, **67**, (in press).

GRALLA, R.J., ITRI, L.M., PISKO, S.E., SQUILLANTE, A.E., KELSEN, D.P., BRAUN, D.W., BORDIN, L.A., BRAUN, T.J. & YOUNG, C.W. (1981). Antiemetic efficacy of high dose metaclopromide: randomized trials with placebo and procloperazine in patients with chemotherapy induced nausea and vomiting. *N. Engl. J. Med.*, **305**, 905–909.

GRUNBERG, S.M., STEVENSON, L.L., RUSSELL, C.A. & MCDERMED, J.E. (1989). Dose ranging phase I study of the seratonin antagonist GR38032F for prevention of cisplatin-induced nausea and vomiting. *J. Clin. Oncol.*, **7**, 1137–1141.

JOHNSON, R.O., METTER, G., WILSON, W., HILL, G. & KREMENTZ, E. (1976). Phase I evaluation of DTIC (NSC-45388) and other studies in malignant melanoma in the Central Oncology Group. *Cancer Treat. Rep.*, **60**, 183–187.

MITCHELL, E.P. & SCHEIN, P.S. (1984). Gastrointestinal toxicity of chemotherapeutic agent. In: *Toxicity of Chemotherapy*, Perry, M.C. & Yarbro, J.W. (Eds). pp. 269–295. Grune & Stratton: Orlando.

MOORE, G.E. & MEISELBAUGH, D. (1976). DTIC (NSC-45388) toxicity. *Cancer Treat. Rep.*, **60**, 219.

PRITCHARD, K.I., QUIRT, I.C., COWAN, D.H., OSOBA, D. & KUTAS, G.J. (1980). DTIC therapy in metastatic malignant melanoma: a simplied dose schedule. *Cancer Treat. Rep.*, **64**, 1123–1126.

STABLES, R., ANDREWS, P.L.R., BAILEY, H.E., COSTALL, B., GUNNING, S.J., HAWTHORN, J., NAYLOR, R.J. & TYERS, M.B. (1987). Antiemetic properties of the 5HT3-receptor antagonists, GR38032F. *Cancer Treat. Rev.*, **14**, 333–336.

WHO Handbook for Reporting Results of Cancer Treatment: World Health Organization, *WHO Offset Publication* No. 48, Geneva, 1979.